# Acute Otitis Media and Facial Paralysis in Children: A Systemic Review and Proposal of an Operative Algorithm

**DOI:** 10.3390/audiolres13060077

**Published:** 2023-11-08

**Authors:** Piergabriele Fichera, Luca Bruschini, Stefano Berrettini, Silvia Capobianco, Giacomo Fiacchini

**Affiliations:** Department of Surgical Pathology, Medical, Molecular and Critical Area, ENT Section, Pisa University Hospital, Via Paradisa, 2, 56126 Pisa, Italy; luca.bruschini@unipi.it (L.B.); s.berrettini@med.unipi.it (S.B.); silviacapobianco.md@gmail.com (S.C.); g.fiacchini@gmail.com (G.F.)

**Keywords:** AOM, facial palsy, pediatric facial palsy, acute otitis media, middle ear inflammation, facial paralysis

## Abstract

Acute otitis media (AOM) is one of the most common ENT diseases in children. In the antibiotic/post-antibiotic era, facial paralysis is a very rare complication of AOM (0.004–0.005%). Despite the rarity of this complication, it should be known by all physicians for proper therapeutic management to avoid serious sequelae. The aim of this review is to provide a management guide based on the current literature. Materials and Methods: Fifteen studies published between 2000 and 2022 were selected, including 120 patients (62 M/58 F) with an average age of 4.96 years old (range = 4 months–16 years; SD: 4.2). The paralysis frequently has a sudden onset and is of a severe grade (medium House–Brackmann (HB) score at onset: 4.68; SD: 0.5); however, it tends to have an almost complete recovery in most patients (88.49% HB 1 at follow-up). Results: Its first-line treatment must be based on the use of antibiotics (beta-lactam antibiotics as penicillins or cephalosporins). Corticosteroids should be used concomitantly for their anti-inflammatory and neuroprotective actions; however, there is no unanimity between authors about their application. Myringotomy, with or without ventilation tube insertion, is indicated in cases where the tympanic membrane is intact. Other kinds of surgery should be performed only in patients who have a worsening of their AOM symptoms or a worsening in HB score even with clinical treatment. Conclusions: The obtained data show that a conservative treatment can be sufficient for complete recovery in most patients, and it is preferred as the first-line therapy. Mastoidectomy should be performed only in patients with acute mastoiditis and without symptom improvement after a conservative approach. There are insufficient data in the current literature to provide clear selection criteria for patients who need to undergo mastoidectomy with facial nerve decompression. The choice of this treatment is based on an individual center expertise. Further studies are needed to clarify the role of corticosteroids and the role of facial nerve decompression in this clinical scenario.

## 1. Introduction

Acute otitis media (AOM) is the most commonly diagnosed childhood disease; approximately 80% of children have at least one episode of AOM before reaching school age [1]. It is characterized by middle ear effusion, ear pain, and/or fever [2], and it is usually preceded by an upper respiratory tract infection or “common cold” [2,3]. The condition is predominantly a bacterial disease, with bacteria cultured in 62% of patients; however, about 5% of patients have ear fluid samples that show viruses only [2,4,5]. Bacteria such as *Streptococcus pneumoniae*, *Haemophilus influenzae*, and *Moraxella catarrhalis* are commonly linked to AOM, as are opportunistic bacteria such as *Staphylococcus* spp., Diphtheroids, *Streptococcus viridans*, and *Neisseria* spp. [6]. On the other hand, viruses such as respiratory syncytial virus, rhinovirus, influenza viruses, and adenoviruses have been noted in some samples [7]. As reported in 2017 by the “International consensus (ICON) on management of otitis media with effusion in children”, several factors act as risk factors in AOM. Pollution, allergy, gastroesophageal reflux disease (GERD), and genetics have been reported as risk/contributing factors for AOM. However, the most important causal factor in this clinical scenario is the Eustachian tube’s reduced efficiency in children [8]. Complications of AOM, including acute mastoiditis, facial palsy, meningitis, and brain abscesses, are rare, and their incidence is lower than that in the pre-antibiotics era. Nowadays, facial paralysis (FP) is an uncommon complication of acute inflammation of the middle ear, with an estimated incidence of 0.004–0.005% [1,9]. Considering the serious impact of FP on young patients’ quality of life, it is important for all ENT clinicians to provide proper therapeutic management in this rare clinical scenario. The pathophysiology of facial paralysis in the case of AOM is still unclear, and several hypotheses have been put forward. As reported by De Zinis et al. in the early 2000s, FP in AOM may be the result of six different pathogenic pathways: [10] (a) the direct involvement of the facial nerve by infection through bony dehiscences or physiologic canaliculi for neurovascular connections with the middle ear; (b) fallopian canal osteitis with bone erosion and nerve involvement; (c) inflammatory edema leading to compression and secondary thrombosis of the vasa nervorum with consequential ischemia and infarction of the facial nerve; (d) reactivation of a latent neurotropic virus, for example, herpesvirus; (e) demyelination of the facial nerve caused by bacterial toxins; or (f) reduced host immunologic response.

The facial nerve is responsible for facial expression, lacrimation, and salivation. Upon physical examination, a patient with facial nerve palsy is unable to raise their eyebrow or close their eyelid on the affected side. The nasolabial fold is typically absent, and the affected side presents a dropped mouth rim, with possible saliva leakage and the inability to smile [11]. A facial nerve evaluation, in terms of facial movements and spontaneous expressions, should be classified according to the House–Brackmann grading system. The House–Brackmann grading system is the most widely used grading system for FP; it involves a six-point scale with I being normal and VI being total, flaccid paralysis (Table 1) [12]. To date, no treatment consensus on AOM-related facial palsy is available.

## 2. Material and Methods

This review was conducted following the requirements of the Preferred Reporting Items for Systematic Reviews and Meta-Analysis (PRISMA). We performed a review of the literature, looking for studies about AOM with facial palsy in pediatric patients (0–18 years old). We searched the PubMed and Cochrane libraries by combining the following words: acute otitis media; middle ear acute inflammation; facial palsy; facial paralysis.

We included papers that fulfilled the following inclusion criteria:

− Written in English;
− Studies published between 2000 and 2022;
− Studies about pediatric patients (age: 0–18 years old);
− Studies using House–Brackmann score.

## 3. Results

**Figure 1:**
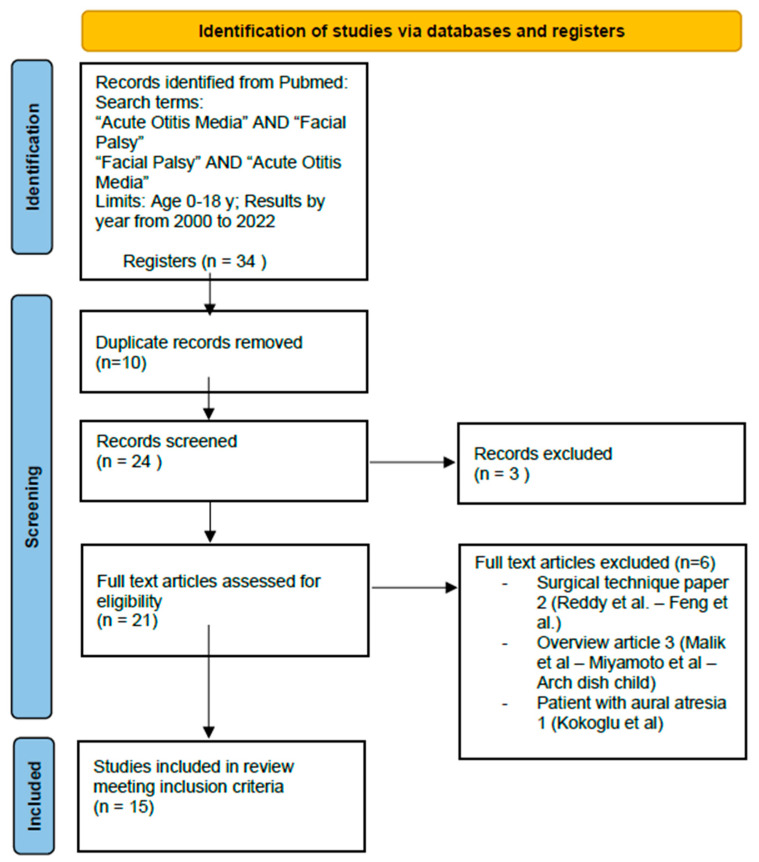
PRISMA. Full text articles excluded: Facial Paralysis in Children—Reddy et al., 2015 doi: 10.1055/s-0035-1549042; Subtotal facial nerve decompression for recurrent facial palsy in Melkersson Rosenthal syndrome—Chuanfu Dai, Shui Feng et al., 2014 doi: 10.3109/00016489.2013.863431; Paediatric facial paralysis: An overview and insights into management—Malik et al., 2021 doi: 10.1111/jpc.15498; Pediatric neurotology—Miyamoto et al., 2003 doi: 10.1016/s1071-9091(03)00070-6; Investigation and treatment of facial paralysis—Arch Dis Child 2001 doi: 10.1136/adc.84.4.286; Acute Otitis Media and Facial Paralysis in an Infant with Aural Atresia: Management of a Rare Case—Kokoglu et al., 2021 doi: 10.5152/JIAO.2021.8569.

Based on the inclusion and exclusion criteria, we were able to retrieve 34 papers. After careful, independent screening by two authors, 15 studies were selected, as shown in Figure 1. Differing opinions were resolved by consensus between the two authors and a third author.

**Figure 2:**
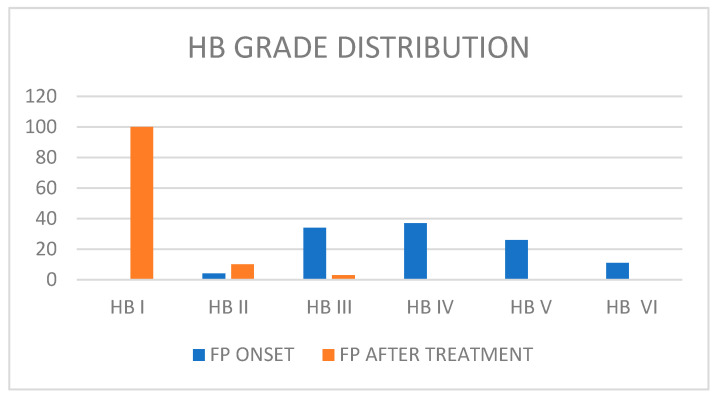
Patients’ facial palsy grades.

The 15 studies included a total of 120 patients (62 M/58 F) with an average age of 4.96 years old (SD: 4.2). We found a medium House–Brackmann (HB) score at onset: 4.68 (SD: 0.5). All available data about patients’ facial palsy grades are reported in Figure 2. Facial nerve paralysis (FNP) secondary to AOM was treated urgently with intravenous antibiotics with or/without steroids. First-line treatment was always based on antibiotics. Amoxicilline was the preferred first-line therapy. Steroids were administrated in 10 studies (71.40% of all patients). Surgery was performed in 70 patients (58.33%). Myringotomy with or without ventilation tube placement was performed in 55 patients (45.8%). It was the most common surgical procedure executed in this clinical scenario, as reported in Table 2. Mastoidectomy was performed in seven patients, while mastoidectomy with facial nerve decompression was executed in eight patients. Detailed information about the follow-up timing was provided by only five studies, with a mean follow-up period of 4.63 months, ranging from a few days to 12 months. In the other hand, information about FP HB grade after therapy was available for 113 patients (94.16% of total). After therapy and follow-up, 100 patients had a complete recovery (88.49%), 10 patients had a final HB score of II (8.84%), and 3 patients had a final HB score of III (2.65%) (Figure 1). Among the group of patients who had a final HB score of III after treatment, two patients received only medical treatment based on systemic antibiotics and corticosteroids and one patient received medical treatment and myringotomy plus ventilation tube placement.

## 4. Discussion

The course of facial nerves is one of the most intricate among cranial nerves. The facial nerve has motor and sensory roots, with the latter being the intermediate nerve. The motor root supplies branches to the muscles of the face, scalp, and auricle; the buccinator; the platysma; the stapedius; the stylohyoid; and the posterior belly of the digastric muscle. The sensory root conveys nervous information to the chorda tympani from the tongue, palatine, and greater petrosal nerve, as well as taste fibers from the soft palate. It is also involved in preganglionic parasympathetic (secretomotor) innervation of the submandibular and sublingual salivary glands, lacrimal gland, and glands of the nasal and palatine mucosa [13]. Due to the facial nerve running through a narrow bony canal within the intratemporal course, any inflammation of or growth in the nerve will result in ischemic changes through compression. The nerve is narrowest at the labyrinthine segment, which extends from the fundus of the internal acoustic meatus to the geniculate ganglion; therefore, compression is most likely to occur at this point [14,15]. The tympanic segment of the facial nerve is another area extremely vulnerable to inflammatory processes within the middle ear. Indeed, the wall of the fallopian canal at this segment is thin, and frequently, there are dehiscences present. Dehiscences are nonpathologic breaches in the continuity of the fallopian canal that allow inflammation of the middle ear to spread to the facial nerve [16]. FP is a clinical complication considered to be due to a bacterial cause although bacteria can be isolated from the middle ear only in approximately two-thirds of cases of AOM [4]. In addition, 5% of the middle ear effusion isolated from children with AOM contain only viruses [17]. From our literature review, Hostetler et al. reported a case about an infant with AOM and FP with cultures of middle ear fluid positive for Coxackie B5 [18]. Vogelnik et al. underlined the role of EBV acute infection in this clinical scenario [4]. Due to its more probable bacterial origin, first-line treatment in AOM + FP should be based on antibiotics. Amoxicillin was the preferred first-line therapy. In fact, amoxicillin should be the initial treatment in the absence of a known allergy [12,19]. Intravenous or intramuscular antibiotics should be reserved for episodes of treatment failure [12,20]. As reported by the Italian Society of Pediatrics ‘Guidelines for Management of AOM’, amoxicillin or cephalosporins should be used as first-line therapy because of their efficacy against the most common bacteria linked to AOM (*Streptococcus pneumoniae*, *Haemophilus influenzae*, and *Moraxella catarrhalis*). These drugs disrupt the synthesis of the peptidoglycan layer forming the bacterial cell wall. Without a cell wall, a bacterial cell is vulnerable to outside water and molecular pressures, which causes the cell to quickly die. Amoxicillin should be used with high dosages (80–90 mg/kg/day), and the daily dosage should be split into 2–3 doses to ensure that adequate concentrations above the MIC are maintained for a sufficiently long time over a 24 h period. Other antibiotics such as Macrolides should only be used in children with a documented history of recent and/or severe allergy to penicillin [21]. Steroids were administrated in 10 studies; however, their use is still controversial [4,16,22]. As reported by the “International consensus (ICON) on management of otitis media with effusion in children” and the “Updated Guidelines for the Management of Acute Otitis Media in Children by the Italian Society of Pediatrics”, the use of systemic corticosteroids should be avoided as first-line treatment in AOM [8,21]. However, their use should be considered in severe or complicated cases. Some authors suggest that the combined use of steroids and antibiotics improve the resolution of middle ear exudates when compared with antibiotics alone [23]. A systematic review by Ranakusuma et al. evidenced an overall improvement in symptoms and a faster resolution of the middle ear inflammation in children treated with systemic steroids [24]. Furthermore, the use of corticosteroid in FP has been advocated by several authors; it seems that steroids may reduce the time of recovery, especially when administered early in the disease course [11,25,26]. On the other hand, some other authors believe that steroids should not be used in the treatment of facial paralysis and may cause serious complications such as adrenal suppression, peptic ulcer disease, and increased susceptibility to infection [16]. It is not possible to define the impact of steroid use in this clinical scenario from our data; however, according to our clinical experience, their use should be considered for their anti-inflammatory and neuroprotective actions. Myringotomy allows for aspiration of middle ear exudates through the tympanic membrane, thus liberating the facial nerve from inflammation and pressure. The ventilating tube prevents early closure of the tympanic membrane. At the same time, the grommet plays a role in the continued drainage of the middle ear; concomitant acute mastoiditis is thus prevented [27]. Myringotomy should be used as a diagnostic tool to collect samples for antibiogram, and it should also help clinicians in the choice of the most appropriate antibiotic regimen [1]. Considering its diagnostic and therapeutic capacity, we support this surgical procedure in this clinical scenario as first-line treatment associated with the use of antibiotics. Moreover, myringotomy with/without a ventilation tube seems to be all that is required for the majority of patients affected by AOM + FP [9,11,27,28,29]. CT scans have been performed in all patients subjected to a conservative treatment without any clinical improvement. A CT scan is the radiologic study of choice; it allows for other pathologies, malformations, and intracranial complications to be excluded, and it is useful to plan additional surgical intervention [1,27,29]. Some authors suggest that CT scans must be executed as soon as possible in all patients with AOM and FP [16]. On the other hand, other authors state that CT scans must be reserved for patients in whom paralysis does not improve within 7 days of onset of a conservative therapy [1]. According to our data, we cannot suggest an optimal time frame to execute a CT scan; however, we support the use of CT scan as a first choice exam for patients without clinical improvement after conservative therapy. MRI scanning is useful for the detection of intratemporal lesions that may resulting the compression of the facial nerve and for imaging of the cerebellopontine angle, and it is also very accurate in identifying an enhancement in the facial nerve around the geniculate ganglion [15]. However, MRI imaging is more expansive, more invasive, and less available than CT scans, so some authors suggested that this test is not indicated in the majority of children [30]. According to our literature review, mastoidectomy or mastoidectomy with facial nerve decompression were reserved for a few patients (15 patients—12.5%); in particular, mastoidectomy was performed in patients with acute mastoiditis and in patients with diffuse opacity of the middle ear and mastoid cavities [1,4,27,31], while mastoidectomy with facial nerve decompression was executed in eight patients [9]. During surgery, some areas of bone dehiscence and edema of the tympanic portion of the facial nerve have been observed in patients who underwent mastoidectomy with nerve decompression supporting, the theory of infectious involvement of the facial nerve happening through the bony dehiscence and neurovascular communication between the middle ear and facial nerve [9]. Patients who need facial nerve decompression were selected through an electrophysiological test in the study by Gaio et al. [1]. Electroneurography (ENoG) was performed between days IV and XIV, and patients with >95% documented degeneration underwent surgery. Electroneuronography (ENoG) is useful in pediatric patients to select the ones expected to have poor functional outcome [1,15]. Other factors suggestive of a poor prognosis when associated with an FP include complete palsy/high HB score, a loss of stapedial reflex, no signs of recovery after 3 weeks, and Ramsey Hunt Syndrome [15,26,27]. All eight patients treated with nerve decompression had an FP clinical improvement [9]. Despite these positive results, the literature still considers mastoidectomy with facial nerve decompression controversial, and some authors suggest that only mastoidectomy is indicated [22,28]. Complete recovery was observed in 88.49% patients of the cohort. Considering that invasive surgical treatment was needed just for a small group of patients (15), we can state that a conservative treatment is preferred and should be sufficient in most cases of AOM and FP.

**Figure 3:**
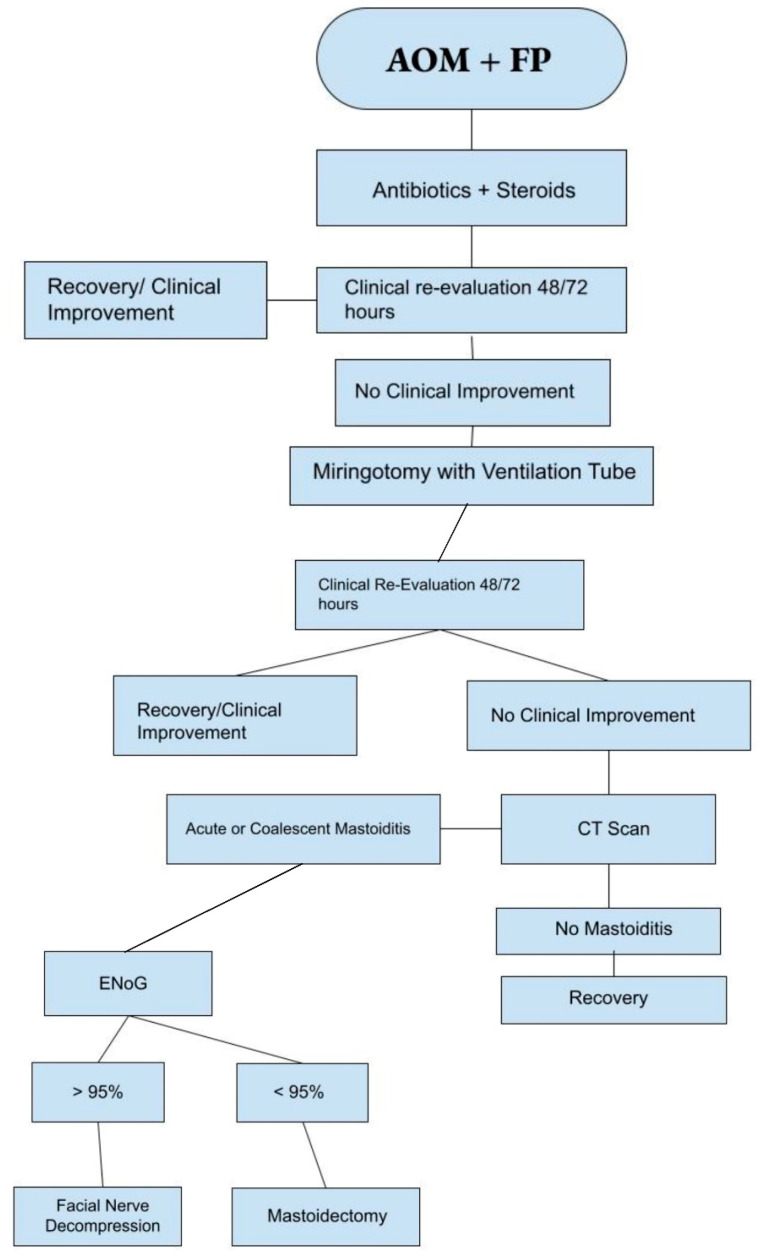
Suggested management algorithm for AOP + FP.

We suggest a practical algorithm based on the literature data and our clinical experience (Figure 3) that should be adopted as a guide in the management of this clinical scenario.

## 5. Conclusions

Facial paralysis is a very rare complication of AOM. First-line treatment should always be conservative and based on the use of antibiotics (specially on beta-lactam antibiotics as penicillins or cephalosporins). Corticosteroids should be used concomitantly for their anti-inflammatory and neuroprotective actions. However, there is no unanimity between authors about their application, and further studies are needed to clarify their possible role in this scenario. Myringotomy, with or without ventilation tube insertion, must be performed as the first-choice surgical procedure due to its diagnostic and therapeutic actions. Conservative treatment can be sufficient for complete recovery in most patients. Other kinds of surgery should be performed only in patients who have a worsening of their AOM symptoms or a worsening in HB score even with clinical treatment. Further studies are needed to clarify the role of mastoidectomy with facial nerve decompression in this clinical scenario.

## 6. Limitations

The present study was subjected to all limitations of work surrounding rare clinical scenarios. As FNP in pediatric AOM is a very rare condition, we included in our work a small number of studies and patients. In addition, due to lack of specific data, it was not possible to determine what kinds of surgical treatment were received by nine of the patients with a final HB grade of II and the effectiveness of mastoidectomy plus facial nerve decompression in terms of FNP grade improvement. Although this study has its limitations, it is hoped that it can serve as a basis for further study in clarifying the optimal clinical management of this rare clinical scenario.

## Figures and Tables

**Table 1:** House–Brackmann facial nerve grading system.

Grade	Description	Clinical Features
I	Normal	No paresis
II	Mild paresis	No abnormalities at rest
III	Moderate paresis	No deformity at rest. Facial synkinesis. With maximum effort, the patient can completely close their eyelids.
IV	Moderate–severe paresis	Obvious asymmetry. Facial synkinesis. Even with maximum effort, the patient cannot completely close their eyelids.
V	Severe paresis	Obvious asymmetry at rest (ptosis of labial commissure, disappearance of the nasolabial fold). Incomplete eyelid closure. Asymmetry in mouth motion.
VI	Complete paralysis	Atony at rest. No facial function.

**Table 2:** Surgical procedures and number of patients.

Surgical Procedures	Patients (%)
Miringotomy +/− Ventilation tube	55/120 (45.83%)
Mastoidectomy	7/120 (5.83%)
Mastoidectomy with facial nerve decompression	8/120 (6.66%)

## References

[1] Gaio E., Marioni G., de Filippis C., Tregnaghi A., Caltran S., Staffieri A. (2004). Facial nerve paralysis secondary to acute otitis media in infants and children. Child Health.

[2] Venekamp R.P., Schilder A.G.M., Heuvel M.v.D., Hay A.D. (2020). Acute otitis media in children. BMJ Clin. Updates.

[3] Chonmaitree T., Alvarez-Fernandez P., Jennings K., Trujillo R., Marom T., Loeffelholz M.J., Miller A.L., McCormick D.P., Patel J.A., Pyles R.B. (2015). Symptomatic and asymptomatic respiratory viral infections in the first year of life: Association with acute otitis media development. Clin. Infect. Dis..

[4] Vogelnik K., Matos A. (2017). Facial nerve palsy secondary to Epstein–Barr virus infection of the middle ear in pediatric population may be more common than we think. Wien. Klin. Wochenschr..

[5] Chonmaitree T., Ruohola A., Hendley J.O. (2012). Presence of viral nucleic acids in the middle ear: Acute otitis media pathogen or bystander?. Pediatr. Infect. Dis. J..

[6] Sakulchit T., Goldman R.D. (2017). Antibiotic therapy for children with acute otitis media. Can. Fam. Physician Clin. Updates.

[7] Simon F., Haggard M., Rosenfeld R., Jia H., Peer S., Calmels M.-N., Couloigner V., Teissier N. (2018). International consensus (ICON) on management of otitis media with effusion in children. Eur. Ann. Otorhinolaryngol. Head Neck Dis..

[8] Yonamine F.K., Tuma J., da Silva R.F.N., Soares M.C.M., Testa J.R.G. (2009). Facial paralysis associated with acute otitis media. Braz. J. Otorhinolaryngol..

[9] de Zinis L.O.R., Gamba P., Balzanelli C. (2003). Acute Otitis Media and Facial Nerve Paralysis in Adults. Otol. Neurotol..

[10] Castellazzi M.L., Torretta S., Di Pietro G.M., Ciabatta A., Capaccio P., Caschera L., Marchisio P. (2023). Acute otitis media-related facial nerve palsy in a child: A case report and a literary review. Ital. J. Pediatr..

[11] House J.W., Brackmann D.E. (1985). Facial nerve grading system. Otolaryngol. Head Neck Surg..

[12] Harmes K.M., Blackwood R.A., Burrows H.L., Cooke J., Van Harrison R., Passamani P.P. (2013). Otitis Media: Diagnosis and Treatment. Am. Fam. Physician.

[13] Yang S.H., Park H., Yoo D.S., Joo W., Rhoton A. (2020). Microsurgical Anatomy of the Facial nerve. Clin. Anotomy.

[14] Walker N.R., Mistry R.K., Mazzoni T. (2023). Facial Nerve Palsy.

[15] White N., McCans K. (2000). Facial paralysis secondary to acute otitis media. Pediatr. Emerg. Care.

[16] Schilder A.G.M., Chonmaitree T., Cripps A.W., Rosenfeld R.M., Casselbrant M.L., Haggard M.P., Venekamp R.P. (2016). Otitis media. Nat. Rev. Dis. Primers.

[17] Hostetler M.A., Suara R.O., Denison M.R. (2002). Unilateral facial paralysis occurring in an infant with enteroviral otitis media and aseptic meningitis. J. Emerg. Med..

[18] Lieberthal A.S., Carroll A.E., Chonmaitree T., Ganiats T.G., Hoberman A., Jackson M.A., Joffe M.D., Miller D.T., Rosenfeld R.M., Sevilla X.D. (2013). The Diagnosis and Management of Acute Otitis Media. Pediatrics.

[19] Bradley M., Bacharouch A., Hart-Johnson T., Burrows H.L., Blackwood R.A. (2021). Adopting otitis media practice guidelines increases adherence within a large primary care network. J. Paediatr. Child Health.

[20] Marchisio P., Galli L., Bortone B., Ciarcià M., Motisi M.A., Novelli A., Pinto L., Bottero S., Pignataro L., Piacentini G. (2019). Updated Guidelines for the Management of Acute Otitis Media in Children by the Italian Society of Pediatrics. Pediatr. Infect. Dis..

[21] Popovtzer A., Raveh E., Bahar G., Oestreicher-Kedem Y., Feinmesser R., Nageris B.I. (2005). Facial palsy associated with acute otitis media. Otolaryngol.—Head Neck Surg..

[22] Shokri T., Saadi R., Schaefer E.W., Lighthall J.G. (2020). Trends in the Treatment of Bell’s Palsy. Facial Plast. Surg..

[23] Ranakusuma R.W., Pitoyo Y., Safitri E.D., Thorning S., Beller E.M., Sastroasmoro S., Del Mar C.B. (2018). Systemic corticosteroids for acute otitis media in children. Cochrane Database Syst. Rev..

[24] Malik M., Cubitt J.J. (2021). Paediatric facial paralysis: An overview and insights into management. J. Paediatr. Child Health.

[25] Wohrer D., Moulding T., Titomanlio L., Lenglart L. (2022). Acute Facial Nerve Palsy in Children: Gold Standard Management. Children.

[26] Psillas G., Antoniades E., Ieridou F., Constantinidis J. (2018). Facial nerve palsy in children: A retrospective study of 124 cases. J. Paediatr. Child Health.

[27] Jacobsson M., Tjellstrom A. (1990). Acute Otitis Media and Facial Palsy in Children. Acta Prediatr. Scand..

[28] D’Anna C., DIplomatico M., Tipo V. (2018). Facial palsy in a baby with acute otitis media. Arch. Dis. Child. Educ. Pract. Ed..

[29] Riordan M. (2001). Investigation and treatment of facial paralysis. Arch. Dis. Child.

[30] Hydén D., Åkerlind B., Peebo M. (2006). Inner ear and facial nerve complications of acute otitis media with focus on bacteriology and virology. Acta Oto-Laryngol..

[31] Evans A.K., Licameli G., Brietzke S., Whittemore K., Kenna M. (2005). Pediatric facial nerve paralysis: Patients, management and outcomes. Int. J. Pediatr. Otorhinolaryngol..

